# Postoperative Osteopathic Manipulative Treatment in Children with Esophageal Atresia: Potential Benefits on the Anthropometric Parameters

**DOI:** 10.3390/pediatric14040051

**Published:** 2022-10-20

**Authors:** Andrea Manzotti, Alessia Alati, Matteo Galli, Francesco Cerritelli, Chiara Leva, Adele Alberti, Alessandro Stizzoli, Sara Costanzo, Carlotta Paola Maria Canonica, Francesca Destro, Gianvincenzo Zuccotti, Valeria Calcaterra, Gloria Pelizzo

**Affiliations:** 1 RAISE Laboratory, Clinical Based-Human Research Department, Foundation COME Collaboration, 65121 Pescara, Italy; 2 Division of Neonatology, “V. Buzzi” Children’s Hospital, ASST-FBF-Sacco, 20154 Milan, Italy; 3 Research Department SOMA, Istituto Osteopatia Milano, 20126 Milan, Italy; 4 UOC of Pediatric Surgery Unit, Children’s Hospital “V. Buzzi” ASST-FBF-Sacco, 20154 Milan, Italy; 5 Pediatric Department, Children’s Hospital “Vittore Buzzi”, 20154 Milan, Italy; 6 Department of Biomedical and Clinical Sciences “L. Sacco”, University of Milan, 20157 Milan, Italy; 7 Pediatrics and Adolescentology Unit, Department of Internal Medicine, University of Pavia, 27100 Pavia, Italy

**Keywords:** esophageal atresia, osteopathic manipulative treatment, pediatric surgery, children

## Abstract

Esophageal atresia (EA) is a congenital malformation that affects the normal esophageal development. Surgical treatment, although restoring the integrity of the alimentary tract, may lead to long-term sequelae-like developmental abnormalities and musculoskeletal deformities. We evaluated the effects of osteopathic manipulative treatment (OMT) on the recovery of the range of the right upper limb movement and on the rise of the auxological parameters. A case series of five children affected by type C EA were described. Six OMT sessions were performed over a 4-month period. At each treatment, height, weight, body mass index (BMI) and range of motion (ROM) in elevation of the right upper limb were assessed. OMT was applied to improve scar, larynx, rib cage, and sternum mobility. An average change of 2.3 cm in height and an average increase of 8° in the ROM of the upper limb in the period of study were detected. Additionally, OMT could improve the anthropometric data and the mobility of the right upper limb of children surgically treated for EA. Further studies that evaluate the effectiveness of OMT in post surgical treatment of congenital malformations of the thorax can be considered in the future.

## 1. Introduction

Esophageal atresia (EA) is represented by the congenital absence of a portion of the esophagus. It is considered a rare disease, with an incidence of 1 in 2500 births [1]. According to the Gross classification (Esophageal atresia with a distal tracheoesophageal fistula), type C is the most frequent form, accounting for 85% of all cases [1,2]. Infants affected by this pathology usually undergo surgery during the early stages of life, and the intervention may be performed through a thoracotomy or a thoracoscopy, depending on the patient’s characteristics and the surgeon’s choice.

A posterolateral thoracotomy is frequently used in pediatric surgery. It provides an adequate working space and access to the posterior mediastinum, allowing for expansion of the surgical field as needed in cases of technical difficulties or complications [3,4,5]. Through this incision, the surgeon cuts at least one (often the latissimus dorsi) or several major thoracic muscles such as the anterior dentate, trapezius, and rhomboid muscles [4].

This type of surgery may lead to severe complications such as acute postoperative pain, disturbance to lung dynamics, and a reduction in the performance of the right shoulder girdle [3]. In the long term, this surgical approach can generate comorbidities affecting the musculoskeletal system, such as the alteration of the scapulohumeral kinetics, morphological dysfunction of the spine such as thoracogenic scoliosis, and alterations of the respiratory kinetics, such as costal fusions [4]. The cause of these sequelae could be attributable to post-surgical scar adhesions, which reduce the resilience of the tissue–including the fascial connective tissue–and its elastic return capacity if distorting forces are applied. Fascial tissue is connective tissue that surrounds blood vessels, nerves, viscera, meninges, bones, and muscles, dividing the body into various layers at different depths [6]. Surgery, as is the case with trauma or diabetes, can alter fascial tissue favoring fibrosis [7].

Osteopathy is a complementary alternative medicine (CAM) based on manual evaluation and treatment. “Touch” has a fundamental role in osteopathy, both in the assessment of the patients [8] and for treating different parts of the body such as the fascial system, following the World Health Organization (WHO) benchmark for the osteopathic profession [9,10]. Several studies have shown how OMT can modulate the inflammatory cytokines leading to the regulation of fibroblast activity, and probably a large-scale relaxation of the connective tissue [11,12,13]. OMT can also improve the range of motion (ROM), demonstrated in different clinical contexts [6,14,15] by using different types of techniques including myofascial release, articular and visceral ones. A gentle approach to the musculoskeletal structure appears to be helpful to modulate the activity of the autonomic nervous system (ANS) [16,17]. An increase of the parasympathetic function by interacting with the interoceptive process at the brain level is apparent, specifically the insular cortex, and its control on the whole body [9,18,19]. Reducing the signs of allostatic load in children after surgery is an important condition to prevent postural and neurodevelopmental issues, allowing them to have normal breathing and feeding parameters [20,21,22]. Furthermore, osteopathy has proved to be effective and safe in the pediatric field and in the management of EA [23,24].

In this study, we present a case series of five children operated on for EA type C in neonatal age, in which we describe a possible variation on the right shoulder range of motion (ROM) and other biometric indicators (e.g., BMI, weight and height) after OMT intervention.

## 2. Materials and Methods

### 2.1. Patients

A series of five patients (4 males and 1 female), submitted to surgery for EA type C at the Pediatric Surgery Unit in the “Vittore Buzzi” Children’s Hospital were studied. Demographic, anamnestic, clinical and instrumental data were recorded.

The study was performed according to the Declaration of Helsinki, and was approved by the local ethics committee (563-04/05/2018). Informed written consent was obtained from the parents and/or legal guardian after receiving information about the study.

### 2.2. Clinical Findings and Diagnostic Assessment

After obtaining parental consent, we organized 6 sessions of OMT to be carried out over 4 months. Before each session, the following parameters were assessed: weight, height, body mass index (BMI, calculated as Weight/Height^2^) and the mobility and range of motion (ROM) of the upper limb in elevation through a manual goniometer evaluation. Each child was investigated carefully through osteopathic manual assessment, which consists of the evaluation of resistance in passive motion and in individualization of non-homogenous and non-compliant areas, also known as somatic dysfunction [8,25]. The manual assessment monitors the relevance of the scar limitation at the level of the other areas of the body, and assesses the presence of different regions of restriction on the hyoid, larynx, esophagus/sternum, and ribs/diaphragm. These elements are involved both in the intervention and the malformation itself. The evaluation was performed by an osteopath with at least five years of pediatric experience, through osteopathic procedures described by Bergna et al. (2020) and Manzotti et al. (2020) [8,25].

### 2.3. Therapeutic Intervention

Osteopathic manipulative treatment is used to alleviate somatic dysfunction and thereby restore normal motion and function throughout the body [26].

The main focus of the treatment is to restore a physiological ROM by increasing the movement of the scars, ribs, sternum, vertebrae, superficial cervical fascia, hyoid, larynx, diaphragm and the upper limb. The techniques adopted are all classified in the Glossary of osteopathic terminology. These included joint mobilization, soft tissue techniques, myofascial release, balanced ligamentous techniques (BLT), osteopathy in the cranial field and visceral techniques [27,28]. There was no specific protocol for the choice of techniques; nevertheless, each case was adapted with the most suitable approach.

The second goal of the study was to improve respiratory and swallowing capacity. These functions were frequently impaired in all children with musculoskeletal restrictions.

The right lateral posterior scars were the main restricted areas we focused on during all treatments.

Direct and indirect techniques according to the “black box” method were applied [6] to make the areas of restriction more compliant and free [29], according to the assumption that the manipulative osteopathic treatment supports the metabolic processes, promotes vascularization and improves ROM [6,15,30].

## 3. Results

### 3.1. Patient Data

#### 3.1.1. Case 1

A 5-year-old boy, born at term by eutocic delivery, underwent a right lateral thoracotomy with muscle sparing approach on day 2 of life after a diagnosis of EA with distal fistula. He needed two endoscopic dilatations following the intervention. The child often experienced reflux issues that were treated with specific medications. Respiratory tract infections occurred more than three times per year. He sometimes experienced dysphagia for solid food. His parents reported no significant problems in his daily life activities.

He underwent 6 treatments in 70 days. During this period there was a 3.5 cm increase in his height, going from 106 to 109.5; while the ROM of the upper limb increased from 165° to 171°. On the other hand, a decreased BMI was observed due to a reduction in body weight, Table 1.

#### 3.1.2. Case 2

The second case is a girl of 5 years of age, born by cesarean delivery. A right muscle-sparing lateral thoracotomy was performed at two days of life after a diagnosis of EA with distal fistula. She also presented with a left superior vena cava draining into the left atrium, and sacralization of the first coccygeal vertebra with absence of the others. The girl often experienced gastroesophageal reflux, which was treated through specific drugs and modified postures for overnight rest. Respiratory infections, more than three times a year, were reported.

She received 6 treatments in 77 days. There was a height growth of 3 cm from 99 cm to 102 cm. The ROM of the upper limb remained at 158°. The weight remained constant at 14.7 kg, with a consequent reduction in the BMI, Table 1.

#### 3.1.3. Case 3

A 3-year-old boy, preterm delivered, underwent a right lateral thoracotomy with a muscle sparing technique 9 months after birth for EA type C. He underwent 3 endoscopic dilations after surgery for esophageal stenosis. The child rarely experienced gastroesophageal reflux and did not need any specific drugs. He reported a maximum of three episodes of respiratory infections per year and did not show any problems in eating. He received 3 treatments in 62 days. There was no improvement in body measures, but there was a significant improvement in the ROM of the upper right limb from 137° to 158°.

The intervention was interrupted after 3 treatments due to family problems (difficulties in scheduling further appointments), Table 1.

#### 3.1.4. Case 4

A 3-year-old boy, born by vaginal delivery, small for gestational age (SGA) with an Intrauterine Growth Restriction (IUGR), was diagnosed at birth with esophageal atresia with tracheoesophageal fistula. He was surgically treated through a right muscle-sparing lateral thoracotomy two days later. Respiratory failure occurred after surgery. A fundoplication was performed one year and 7 months after birth for gastro-esophageal reflux after not responding to medical treatment. He also underwent 8 endoscopic dilations during the routine follow-up. He had more than three episodes a year of respiratory infections, followed a specific diet, with frequent symptoms of esophageal food blockage.

The patient underwent 6 treatments in 85 days. His height increased from 90 to 93 cm, his weight increased by 800 g, with stability of BMI. The mobility of the upper limb in elevation raised from 164° to 167° without recording alterations in the normal functions of daily life, Table 1.

#### 3.1.5. Case 5

A 7-year-old boy, born from eutocic birth at 39 weeks of gestation, underwent a lateral thoracotomy with a muscle sparing approach the day after birth, for a diagnosis of esophageal atresia with tracheoesophageal fistula. He sometimes experienced reflux episodes, which were treated with specific drugs. He manifested fewer than three episodes of respiratory tract infections per year. The patient received 4 treatments in less than a month. We recorded a 2 cm increase in height from 115.5 to 117.5, and the upper limb gained in elevation from 150° to 160°. The weight remained constant. The intervention was interrupted after 4 treatments because of the arrival of the summer holiday break, Table 1.

Overall, the median time from thoracotomy to the first osteopathic treatment was 5 years (range 2.25–7 years; mean 4.45 ± 1.68 years).

Table 1 shows pre- and post-treatment biometric parameters values for all cases.

**Figure 1:**
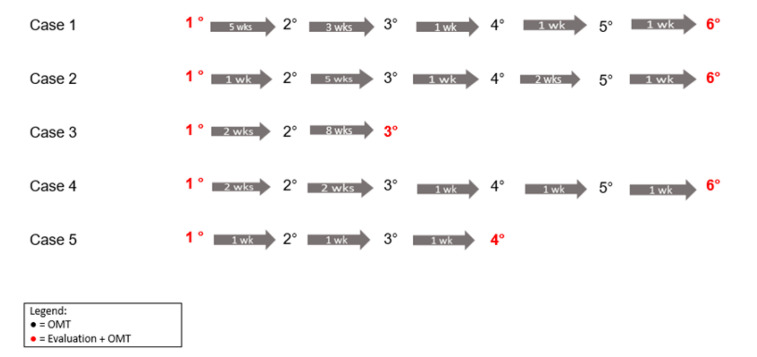
Timeline of osteopathic evaluations and treatments. Numbers indicate the session and arrows show the time between sessions in weeks. OMT = osteopathic manipulative treatment.

The timeline of osteopathic evaluations and treatments of the patients are represented in Figure 1.

##### Technique

In Table 2, the techniques per each case during all OMT sessions are reported.

##### Outcome

As reported in Table 1, changes in the anthropometric parameters were detected.

The treatment showed no adverse events in any of the cases treated. On one occasion, an episode of reflux occurred during the session of OMT, and was resolved by lifting the patient’s head from the treatment table. No other side effects occurred during treatments or were reported by parents.

Regions with an impaired function in each case in the pre- and post-treatment period are described in Table 3.

## 4. Discussion

The data collected showed an improvement in the arc of movement in elevation of the right upper limb and an increase in height, even if the treatment was performed years after surgery.

As the literature suggests, pediatric surgical procedures may have short- and long-term effects on children and their families from both a pathophysiological and psychosocial points of view [31]. Considering the post-surgical sequelae on the musculoskeletal system, recent studies support the concept that the less invasive the surgery is, the more anatomical alterations of the movement system are avoided [5,32]. Our results on ROM could suggest that a better management of these frequent side effects can be offered.

In fact, a ROM deficit of the upper right limb is strongly documented in the literature [3]. Many studies consider the onset of scoliosis, thoracic deformities, scapular elevations, and limitation on the right shoulder ROM as a possible long-term postoperative risk [5,32,33]. The increase in mobility in shoulder elevation that we obtained in four out of five of the children, suggests that more research is needed to test the possibility of intervening through manipulative osteopathic treatment on these kinds of sequelae.

Several studies support the feasibility of increasing joint ROM with OMT in different contexts. Sposato & Bjerså [34] in their review investigated the effects of OMT applied to adults who underwent surgery. The results showed an increase of spinal ROM in flexion, extension, and bilateral lateral side bending in patients with lumbar discectomy compared to an exercise control group, and an increase in lateral flexion in patients with thoracotomy. Serra-Añó et al. [15] have shown an efficacy of myofascial relaxation on shoulder mobility in accordance with our results.

Another finding is the increase in height after the treatment period. Birketvedt et al. [35] reported in their study that 15% of children undergoing surgery for OA were classified as stunted. In our study, we can identify an increase in the height measures of three cases treated, compared with the linear measures of growth: in the first case, the child moved from about the 10th to about the 25th percentile; in the second case, the height changed from minus 2 standard deviations (SD)–as compared to the standard population–to near the 10th percentile; and in the fifth case, it varied from the 10th to over the 25th percentile. These results, according to Frongillo et al. [36], suggest the important role of evaluating the effects of an intervention using linear measures of growth. Weight values did not vary significantly, although we did not consider some parameters including fat mass and lean mass. As Weber et al. [37] showed in their study, body composition measurements can be useful to predict clinical outcomes and nutritional status. Even though the role of additional influences such as nutritional or developmental factors on the statural growth are not excluded, these factors (as per the treatment period) usually affect weight rather than height in a short time (we added the information in the text).

Early surgery in children is a significant stress factor, not only from the physical point of view, but also from the cognitive, emotional and psychosocial ones [31]. Height deficit can be related to an increase in the serum concentrations of proinflammatory cytokines and cortisol as a result of the post-surgery condition [38]. Thinking about these connections and correlating the musculoskeletal system–with all its subdivisions, that include the fascial system–we can suppose that impaired growth may be linked to the presence of stress factors. Osteopathic treatment has demonstrated its efficacy in modulating autonomic nervous system function, favoring the enhancement of parasympathetic activity [30,39,40]. In 2019, a study demonstrated a reduction in allostatic biomarkers –evaluation of cortisol, diurnal catecholamines with urine tests, glycated hemoglobin, high-intensity lipoproteins, high reactive protein C sensitivity, blood pressure, BMI and hip-waist index–following osteopathic manipulative treatment [41]. Furthermore, it would be essential to introduce the evaluation of allostatic biomarkers to determine a real correlation between the results of the treatment and their supposed pathophysiological principles.

Additionally, current findings seem to substantiate the possible relationship between allostasis, osteopathic care, scar adhesion of the shoulder and the presence of somatic dysfunctions. This phenomenon can be explained through a physiological mechanism that takes place during the recovery and the formation of new scar tissue, which involves the proliferation of collagen tissue [7,42]. This altered thixotropic condition of the tissue can be assessed manually, by precise manual semeiotics. The latter then can be used to plan osteopathic manual treatment in order to induce specific neurobiological effects. Indeed, the documented anthropotropic effect of OMT could have a role in balancing the neuroinflammation response and tissue healing, acting further on the capacity of adaptation of the fascia and improving shoulder joint motion [30,43]. Further, in this context the bodily restrictions found after the treatments can be considered an adaptation to allostatic processes, and are not related to specific pathologies. In other words, by using a process of self-regulation, the body generates additional neurobiological adaptive strategies to overcome the musculoskeletal function impaired by surgery [25,43]. These strategies could be supported by osteopathy acting on tissue and fascia stiffness, reducing mechanical allostatic load and modulating the response of the autonomic nervous system [30,43].

It could be interesting to reduce the age of osteopathic intervention on children to ensure that the identified altered functions can be treated and resolved in the shortest possible time, in order to avoid chronic effects.

The authors acknowledge that the study has some limitations. Firstly, the concept of “case series” is not well defined and does not reflect a specific research design; according to the literature, and as in our manuscript, a case series should have more than four patients [44]. Moreover, five patients are still a limited number of cases, and a larger sample size should be considered to determine the absolute efficacy and to define the significance of the improvement in anthropometric parameters. Secondly, the heterogeneity of the patients in terms of age at the beginning of treatment, postoperative surgeries and associated complications could impact on the results. A control group for comparison should also be included in future research to confirm these preliminary findings. Additionally, despite the changes in ROM which has been usually described during the progress through adolescence and growth spurts than during childhood, the influence of physiological growth on the beneficial effects could be not excluded [45,46].

The treatments were conducted according to the “black box” method, well-described and accepted in osteopathy, which involves the application of different techniques depending on the somatic dysfunctions found. This is at the discretion of the operator, and could influence a less than positive final outcome in all patients and in the reproducibility of the results.

Furthermore, documenting the outcomes through photos of the subject on the frontal and sagittal plane, and pictures related to the mobility of the skeletal structures, could certainly improve the quality of the outcome measurements. Another limitation also relates to the timing of OMT after surgery; in fact, this high latency may negatively influence the effect of OMT, which is probably more useful in the early recovery of scar tissue [7].

Further research should also include allostatic biomarkers to better investigate the possible effects on growth and patient reported outcomes, such as quality of life, pain or disability and other measures, i.e., respiratory indicators (e.g., spirometry measures or optoelectronic plethysmography), to investigate other possible related effects.

Despite these limitations, our preliminary experience support shows that osteopathic treatment can be a feasible, well-tolerated and potentially advantageous approach in children with EA submitted to surgery, and which can undergo significant osteo-articular sequelae influencing the anthropometric parameters.

## 5. Conclusions

Our study supports the hypothesis that OMT may be useful to increase the ROM in children operated on with type C EA. The present case series might open doors for the use and development of manual therapy studies and routine healthcare assistance in pediatric surgery, creating opportunities for future multidisciplinary collaborations.

Other studies with a larger sample size are needed to confirm these preliminary effects on the linear measure of growth.

## Figures and Tables

**Table 1:** Synthesis of the biometric values for all the cases studied.

	Case 1		Case 2		Case 3		Case 4		Case 5	
Age (yrs)	5		5		3		3		7	
Parameter										
Pre and post treatment	Pre	Post	Pre	Post	Pre	Post	Pre	Post	Pre	Post
Height (cm)	106	109.5	99	102	86	86	90	93	115.5	117.5
Weight (kg)	18	17	14.5	14.7	10.5	10	12	12.8	22.2	22
BMI	16.02	14.31	14.75	13.46	13.52	13.52	14.81	13.87	16.64	16.07
Right shoulder elevation ROM (°)	165°	171°	158°	158°	137°	158°	164°	167°	150°	160°
Linear measure of growth (height, percentile)	10th	25th	−2 SD to st. pop.	10th	5th	5th	5th	5th	10th	25th
Z-score BMI	0.48	−1.21	−0.29	−0.92	−1.73	−2.55	−1.06	−1.07	0.65	0.25
Percentile BMI	68th	11th	39th	18th	4th	1st	14th	14th	74th	60th
Z-score Height for age	−0.68	0.07	−1.96	−1.28	−2.58	−2.58	−1.42	−0.60	−1.21	−0.84
Percentile Height for Age	25th	53rd	2.5th	10th	0.5th	0.5th	8th	27th	11th	20th
Z-score Weight for Age	−0.21	−0.67	−1.78	−1.65	−3.12	−3.66	−1.73	−1.09	−0.30	−0.36
Percentile Weight for Age	42nd	25th	4th	5th	0.1st	0.1st	4th	14th	38th	36th

BMI = Body Mass Index; ROM = Range of Motion. BMI, weight and height percentile and z-score (standard deviation) was calculated according to the Center for Disease Control (CDC) growth charts.

**Table 2:** Techniques per each case during all OMT sessions.

Case 1	Fascial mobilization on the scar Osteopathy in cranial field BLT on right scapula and shoulder BLT on diaphragm BLT on sternal area BLT on dorsal region Visceral osteopathy
Case 2	Fascial mobilization on the scar MFR on diaphragm BLT on right scapula and shoulder BLT on diaphragm BLT on sternal area BLT on dorsal region Visceral osteopathy
Case 3	BLT on the scar BLT on sternal area BLT on dorsal region Visceral osteopathy
Case 4	BLT on the scar BLT on dorsal region Visceral osteopathy Osteopathy in cranial field BLT on right scapula and shoulder
Case 5	Visceral osteopathy BLT on right scapula and shoulder Osteopathy in cranial field BLT on diaphragm

BLT = Balanced Ligamentous Tension; MFR= Myo-Fascial Release.

**Table 3:** Region restricted pre- and post-treatment period.

	Region Restricted Pre-Treatment Period	Region Restricted Post Treatment Period
Case 1	Right postero-lateral scar Left hypochondrial region Dorsal region (T5-T9)	Dorsal region (T2-T9) Right iliac region Hypomobility of diaphragm muscle
Case 2	Right postero-lateral scar Hypomobility of diaphragm muscle. Dorsal region (T5-T9)	Hypomobility of diaphragm muscle. Sternal hypomobility Hyoid restriction
Case 3	Right postero-lateral scar Hypomobility of diaphragm muscle Dorsal region (T5-T9)	Right hypochondrial region Right sacro-iliac joint Sternal hypomobility
Case 4	Right postero-lateral scar Upper cervical hypomobility Hypomobility of diaphragm muscle	Right iliac region Upper cervical hypomobility Hypomobility of diaphragm muscle
Case 5	Right postero-lateral scar Hypomobility of right scapula Upper cervical hypomobility	Left pocondral region Right iliac region Hypomobility of diaphragm muscle

## Data Availability

Data collected in the first author’s personal databases and available under request.
